# Reactive Astrocytes in Central Nervous System Injury: Subgroup and Potential Therapy

**DOI:** 10.3389/fncel.2021.792764

**Published:** 2021-12-23

**Authors:** GuiLian Yu, Ying Zhang, Bin Ning

**Affiliations:** Jinan Central Hospital, Cheeloo College of Medicine, Shandong University, Jinan, China

**Keywords:** traumatic brain injury, spinal cord injury, reactive astrocytes, scar-forming astrocytes, astrocyte-targeted therapy

## Abstract

Traumatic central nervous system (CNS) injury, which includes both traumatic brain injury (TBI) and spinal cord injury (SCI), is associated with irreversible loss of neurological function and high medical care costs. Currently, no effective treatment exists to improve the prognosis of patients. Astrocytes comprise the largest population of glial cells in the CNS and, with the advancements in the field of neurology, are increasingly recognized as having key functions in both the brain and the spinal cord. When stimulated by disease or injury, astrocytes become activated and undergo a series of changes, including alterations in gene expression, hypertrophy, the loss of inherent functions, and the acquisition of new ones. Studies have shown that astrocytes are highly heterogeneous with respect to their gene expression profiles, and this heterogeneity accounts for their observed context-dependent phenotypic diversity. In the inured CNS, activated astrocytes play a dual role both as regulators of neuroinflammation and in scar formation. Identifying the subpopulations of reactive astrocytes that exert beneficial or harmful effects will aid in deciphering the pathological mechanisms underlying CNS injuries and ultimately provide a theoretical basis for the development of effective strategies for the treatment of associated conditions. Following CNS injury, as the disease progresses, astrocyte phenotypes undergo continuous changes. Although current research methods do not allow a comprehensive and accurate classification of astrocyte subpopulations in complex pathological contexts, they can nonetheless aid in understanding the roles of astrocytes in disease. In this review, after a brief introduction to the pathology of CNS injury, we summarize current knowledge regarding astrocyte activation following CNS injury, including: (a) the regulatory factors involved in this process; (b) the functions of different astrocyte subgroups based on the existing classification of astrocytes; and (c) attempts at astrocyte-targeted therapy.

## Introduction

In 1856, Rudolf Virchow described for the first time a type of cell with neuron-supportive functions (Virchow, 1856). Then, in 1895, MV Lenhossék proposed the name astrocyte (“Astrocyten”) for this type of neuron-supporting cell (Lenhossék, 1893). Cortical astrocytes originate from radial glia derived from the neuroepithelial cells, radial glial cells originate from the cortical ventricular zone and are characterized by a long basal process that extends from the cortical ventricular zone to the pial surface (Arellano et al., 2021). During embryonic development, radial glial cells generate intermediate glial progenitors *via* asymmetric division, and these progenitors then migrate, proliferate, and finally transform into astrocytes in nerve tissue. After birth, astrocytes are primarily generated through the direct transformation of radial glial cells in the ventricular zone, the migration and development of postnatal progenitors in the subventricular zone, and the symmetrical division of differentiated astrocytes (Levison and Goldman, 1993; Ge et al., 2012; Verkhratsky and Nedergaard, 2018; Abdeladim et al., 2019). NG2 glial cells comprise another possible source of astrocytes (Nishiyama et al., 2016). Here, astrocytes undergo limited migration along with radial glial processes (Jacobsen and Miller, 2003). Astrocytes of different origins are phenotypically diverse, which is a partial manifestation of the heterogeneity of astrocyte morphology and function (Magavi et al., 2012; Tsai et al., 2012; Molofsky and Deneen, 2015). A combination of heredity, development, and phenotype renders astrocytes a truly opportunistic cell with lifelong adaptive plasticity.

Under physiological conditions, astrocytes perform a variety of functions primarily associated with the maintenance of CNS homeostasis, including the formation and maintenance of the blood–brain barrier (BBB) and blood–spinal cord barrier (BSCB), signal transmission across synapses, the maintenance of neuronal function, and metabolic regulation (Molofsky and Deneen, 2015). In a pathological background, however, astrocytes can become activated. The lifelong adaptive plasticity of these cells and the complexity of the disease background determine the diversity of astrocyte subpopulations after injury (Verkhratsky and Nedergaard, 2018). Following CNS insult, activated astrocytes can sequentially display two different histological phenotypes over time, first becoming reactive astrocytes (RAs), and then scar-forming astrocytes (SAs; Hara et al., 2017). This sequential phenotypic change from the resting state to the activated state is referred to as reactive astrogliosis (Zamanian et al., 2012). However, this histological classification method fails to clearly define RAs and SAs as it is neither objective nor quantitative.

In 2017, Hara et al. (2017) were the first to define several RA- and SA-specific marker genes in the mouse. *Plaur*, *Mmp2*, *Mmp13*, *Axin2*, *Nes*, and *Ctnnb1* were classified as RA marker genes, while SA markers included *Cdh2*, *Sox9*, and chondroitin sulfate proteoglycan (CSPG)-related genes, such as *Xylt1*, *Csgalnact1*, *Chst11*, *Pcan*, *Acan*, and *Slit2*. Nevertheless, RAs and SAs both display high expression levels of several proteins, including GFAP, nestin, β-catenin, N-cadherin, and SOX9. As the disease progresses, there is an overlap of RA subpopulations and RAs interact with Col1 and are converted into SAs *via* the integrin/N-cadherin pathway (Hara et al., 2017; Li X. et al., 2020). This research is of great significance to the understanding of SAs, but due to the lack of further research, the function of SA is not yet clear. Recently, Escartin et al. (2021) redefined RAs as ‘astrocytes that undergo molecular, morphological, and functional changes in response to pathological stimuli from surrounding tissue, such as CNS disease, injury, and deleterious experimental manipulation, among others. High GFAP expression levels and cell hypertrophy are considered the minimum criteria for defining RAs (Liddelow et al., 2017).

**Figure 1:**
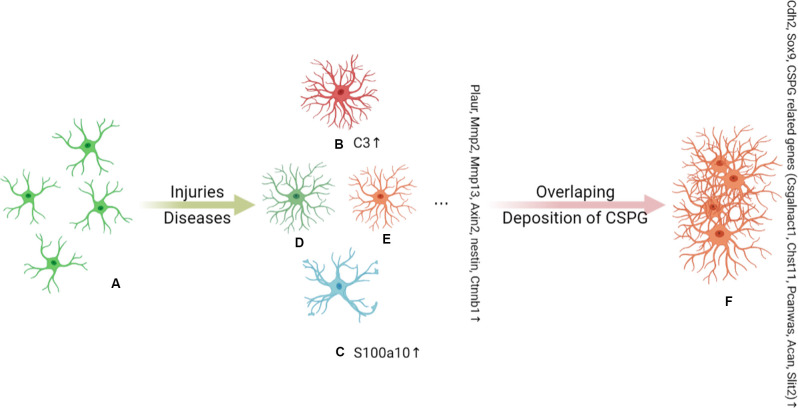
Under the stimulation of injury and disease, **(A)** naive astrocytes are activated into functionally heterogeneous reactive astrocytes (RAs); this heterogeneity is determined by the background of the astrocytes. The *Plaur*, *Mmp2*, *Mmp13*, *Axin2*, *Nes*, and *Ctnnb1* genes are markers of RAs. In an inflammatory background, **(B)** A1 astrocytes are proposed to be a subpopulation of neurotoxic RAs and are marked by C3 expression. **(C)** A2 astrocytes are induced by ischemia and hypoxia and are indicated to play a neuroprotective role in injury and disease. A2 astrocytes can be distinguished by the expression of S100A10. C3^+^ A1 astrocytes have long dendrites, while S100a10^+^ A2 astrocytes have hypertrophic cell bodies with few dendrites. There are other as yet unidentified subpopulations of RAs that also play an important role in disease, such as **(D)** and **(E)**. As the disease progresses, there is an overlap of RA subpopulations and chondroitin sulfate proteoglycan (CSPG) deposits, which together induce the conversion of RAs to SAs **(F)**. *Cdh2*, *Sox9*, and CSPG-related genes (*Csgalnact1*, *Chst11*, *Pcan*, *Acan*, and *Slit2*) are markers of scar-forming astrocytes (SAs).

In addition to the above classification of astrocytes (RAs and SAs), RAs are also divided into different astrocyte subgroups. In 2012, Zamanian et al. undertook a genomic analysis using two mouse injury models (inflammation and cerebral ischemia models) to profile RA phenotypes. The authors found that the RA phenotype was dependent on the type of inducing injury, and identified high Lcn2 and Serpina3n expression levels as strong markers of RA phenotype (Zamanian et al., 2012). In 2017, Liddelow et al. found that neurotoxic RAs, which they named A1 astrocytes, were induced by cytokines (TNF-α, IL-1α, and complement component C1q) secreted by activated microglia, whereas neuroprotective RAs, termed A2 astrocytes, were induced under ischemic and hypoxic conditions. As shown in Figure 1. The neurotoxic effect of complement component 3 (C3), a strong marker of A1 astrocytes, has been confirmed in a variety of CNS diseases, especially the interaction between the C3 cleavage fragment, C3a, and its receptor, C3aR, on neurons (Guo et al., 2010; Lian et al., 2015; Li J. et al., 2020; Yadav et al., 2021). However, the A1 and A2 phenotypes were not proposed to be universal or all-encompassing, they were widely misinterpreted as evidence for a binary polarization of reactive astrocytes in either neurotoxic or neuroprotective states, which could be readily identified in any CNS disease, acute or chronic, like the once-popular, but now discarded, Th1–Th2 lymphocyte and M1–M2 microglia polarization theories. Any binary classification method cannot show the diversity of astrocytes across diseases. More importantly, in mouse models of CNS damage, a RA subset was usually a mixture of A1 and A2 or pan-reactive transcripts (Das et al., 2020). So, Escartin et al. (2021) recommend moving beyond the A1–A2 labels and the misuse of their marker genes. In fact, the latest works of the original authors who studied these subtypes no longer use A1/A2. Guttenplan et al. (2021) used the induction conditions of A1 astrocytes but called the induction results neurotoxic reactive astrocytes. Hasel et al. (2021) used the term neuroinflammatory astrocyte, and used the pattern of “Y-zone X-positive astrocytes showing Z phenomenon” to describe the neuroinflammatory astrocyte subgroups he discovered. Based on existing knowledge, this is an ideal way of naming. However, the A1/A2 classification of RAs is still widely used.

In our opinion, under certain conditions, neurotoxic reactive astrocytes, neuroinflammatory astrocytes, and A1 astrocytes are almost the same. *In vitro*, neurotoxic reactive astrocytes and A1 astrocytes are induced in the same way. In the brain of LPS-induced systemic inflammation mouse model, Liddelow et al proposed the concept of A1 astrocytes, and Hasel et al. proposed various neuroinflammatory astrocyte subtypes, A1 astrocytes can be regarded as a subgroup of neuroinflammatory astrocytes. Neurotoxic reactive astrocytes emphasized function, while neuroinflammatory astrocytes emphasized background, both concepts include A1 astrocytes. At present, users of the A1/A2 concept all regard A1 as the representative of neurotoxic astrocytes and A2 as the representative of neuroprotective astrocytes. However, considering their functional heterogeneity, it is likely that not all neurotoxic RAs are A1 astrocytes, and neither are A2 astrocytes. In the background that current knowledge does not allow objective classification of astrocytes, the use of a binary description of reactive astrocytes (A1/A2, neurotoxicity/neuroprotective), seems unavoidable. Recently, Escartin et al. (2021) reached a consensus that the field should move beyond binary descriptors and embrace objective classification based on their increasingly complex functional heterogeneity. And the work by Liddelow and Hasel supports this view (Hasel et al., 2021).

Astrocytes are key factors in secondary neuronal damage and repair inhibition largely due to their dual role in the regulation of neuroinflammation and glial scar formation after CNS injury (Liddelow and Barres, 2017; Adams and Gallo, 2018). This dual role requires the accurate classification of astrocyte subpopulations. In this review, we will focus on the heterogeneity of astrocytes and astrocyte targeted therapy strategies after CNS injuries (TBI and traumatic SCI) to help the development of targeted therapy strategies based on these precise classification of astrocytes.

## Traumatic CNS Injury

Owing to the preventability of most CNS injuries and the complex and expensive medical care they require, TBI and SCI are increasingly recognized as global health priorities. In 2016, approximately 27.08 million new cases of TBI and 0.93 million new cases of SCI were diagnosed. The age-standardized incidence rate was reported to be 369 per 100,000 population for TBI and 13 per 100,000 for SCI (GBD 2016 Traumatic Brain Injury and Spinal Cord Injury Collaborators, 2019). TBI alone caused annual global economic losses of $US400 billion (Maas et al., 2017). From 1990 to 2016, the age-standardized prevalence of TBI increased by 8.4%, whereas that of SCI did not change significantly. However, given the increase in population density, population aging, and the increased use of motor vehicles, the number of people with SCI is expected to increase. TBI has a higher mortality rate (higher acute injury-related mortality), while TSCI is characterized by a higher standardized mortality rate (shorter long-term life expectancy for SCI survivors; Badhiwala et al., 2019). Public health initiatives to prevent injuries, such as the use of bicycle helmets, fall prevention, policy changes affecting the impact of sports, and other public safety measures, are very effective in reducing the morbidity and mortality associated with TBI and SCI (Taylor et al., 2017). The focus of clinical management involves reducing intracranial pressure, medullary cavity pressure, and cerebral edema, as well as systemic supportive treatment (Maas et al., 2021). In most cases, the effects of these interventions on patients are disappointing (Maas et al., 2017). The burden of disability due to CNS injury can also have a devastating effect on the families of patients because it prevents them from engaging in economic activities.

TBI is divided into focal tissue damage and diffuse tissue damage. Focal injuries are caused by direct impact and include scalp injuries, skull fractures, brain contusions, cerebral hemorrhage, and stroke, which form focal TBI lesions that can vary greatly in size (Gaetz, 2004). Diffuse injury is caused by acceleration–deceleration forces, including hypoxia–ischemic injury, meningitis, and vascular injury (Gaetz, 2004). However, tissue damage after TBI is rarely purely focal or diffuse, and a single case usually involves multiple focal and diffuse lesions (Skandsen et al., 2010). TBI-related tissue pathology and its functional consequences are heterogeneous and determined largely by: (a) the mechanical properties of the injury; (b) the degree of injury severity (mild, moderate, or severe); and (c) the anatomical location of the injury (Burda et al., 2016). The spinal cord has a unique anatomical structure and the impact of scars on the function of the spinal cord at later stages of SCI can be devastating. Consequently, greater attention is given to pathological changes occurring over time. Several key time points are worth noting, such as the 3rd day after injury when inflammation peaks.

Traumatic injury in the CNS is characterized by transient mechanical damage and subsequent delayed non-mechanical damage (Burda et al., 2016). Primary injury in the brain is caused by mechanical force, which immediately leads to contusion and bleeding in the affected area. In the spinal cord, injury usually relates to vertebral fracture or dislocation (Oyinbo, 2011). The secondary injury occurs hours, days, months, or even years after the initial injury, and is characterized by the expansion of tissue damage from the center of the disease. According to the research in the rodent model of TBI, secondary injury can be simply divided into two parts. The first is inflammation, which peaks on the 3rd day after injury (Susarla et al., 2014). Under the stimulation of a wide variety of pro-inflammatory factors produced as a result of the primary injury, microglia and astrocytes are activated, peripheral immune cells are recruited, and the inflammation cascade is initiated. These effects are accompanied by the destruction of the neurovascular unit, glutamate accumulation, oxidative stress, axonal damage, and neuronal death (Gyoneva and Ransohoff, 2015). The second part involves scar formation, in which glial scars begin to form on day 7 post-injury (Villapol et al., 2014). The glial scar surrounds the site of injury and limits the spread of a strong inflammatory response (Burda and Sofroniew, 2014); however, glial scars secrete a variety of cytokines and proteoglycans that promote neurotoxicity and inhibit axon regeneration, respectively (Silver and Miller, 2004). The outcome of glial scarring is the development of a fibrotic scar, which creates a physical and chemical barrier to axon regeneration and nerve function recovery after injury (O’Shea et al., 2017).

The role of an astrocyte is determined by its subgroup status and the surrounding environment. This diversity of astrocyte function directly affects the inflammatory response and glial scar formation after injury. After an injury, astrocytes interact with surrounding cells, such as neurons, microglia, and endothelial cells, that together constitute the post-injury microenvironment, which plays a pivotal role in disease development (Abbott et al., 2006; Valori et al., 2019).

Although primary CNS injuries cannot be treated, secondary injuries provide a therapeutic window for the treatment of the resulting diseases (Wang et al., 2014). Accordingly, to identify effective treatment strategies, research attention has increasingly focused on the role of astrocytes in the pathology of CNS damage.

## Astrocyte Activation After Injury

In response to CNS damage, naïve astrocytes are activated and transform into RAs. This transformation involves changes in morphology, increased expression of the intermediate filament proteins GFAP and vimentin, as well as increased proliferation and secretion of inflammatory mediators and growth factors (Karve et al., 2016). After TBI in mouse, astrocytes react within 24 h and reach a peak of approximately 3–7 dpi, showing a continuous reactive state (Susarla et al., 2014). A recent study conducted using a mouse CCI (chronic constriction injury) model reported the occurrence of astrocyte hypertrophy in the lesion site and surrounding area at 3 days post-injury (dpi). At 7 dpi, the morphological changes became long-lasting, and glial scars began to form (Villapol et al., 2014). In this model, reactive gliosis persisted for up to 60 dpi, indicative of a continuous response of astrocytes to brain injury (Villapol et al., 2014). In another study, after sensorimotor cortex aspiration in adult rats, astrocyte activation lasted for 16 weeks (Basiri and Doucette, 2010).

### Primary Mechanical Stress

In traumatic CNS injury, mechanical stress can cause neuronal membrane instability and cytoskeleton disintegration (LaPlaca et al., 2009). Astrocytes are activated through plasma membrane stretching. The results of a study using astrocytes cultured on deformable membranes indicated that mechanical strain led to AKT activation in astrocytes *via* the stimulation of P2 receptors and promoted ATP release; this, in turn, activated extracellular signal-regulated protein kinase (ERK; Neary et al., 2005). Additionally, the knockout of the Cav1.2 subunit of L-type voltage-operated calcium channels attenuated the migratory and proliferative abilities of astrocytes, indicating that these channels contribute to astrocyte activation, at least *in vitro* (Cheli et al., 2016). In a mouse model of nerve demyelination, reducing voltage-gated Ca2+ influx in astrocytes during brain demyelination significantly attenuated brain inflammation and astrocyte reactivity (Zamora et al., 2020). Indeed, calcium is required for ERK activation in astrocytes, and inhibiting these Ca2+ channels may be an effective means of preventing astrocyte activation and proliferation. In recent research, Hlavac et al showed rat primary astrocytes exposed to high-rate overpressure were mechanically activated, involving changes in structure and junctional proteins (Hlavac and VandeVord, 2019). Their further study indicated that both extracellular adhesion (*via* FAK activation) and cationic conductance (*via* ion channels) contribute to this progress (Hlavac et al., 2020). Wakida et al. (2020) showed astrocyte phagocytosis was a mechanosensitive response, and astrocytes exposed to fluid shear stress initiated phagocytosis at a faster rate than cells observed under static conditions. Liu J. et al. (2021) proposed Piezo1(mechanosensing channel) in astrocytes was involved in the mechanical activation of astrocytes caused by mechanical stretching.

### Secondary Pathological Process

During the secondary pathological process, the release of intracellular components by the cells injured by primary mechanical stress; activation of microglia and astrocytes at the injured site; production of cytokines and chemokines; and recruitment of peripheral immune cells into CNS, these processes influence each other and produce complex interaction. Peripheral cells released signal factors to recruit extra cells from the periphery and maintain the activation of microglia and astrocytes, leading to excessive activation of astrocytes, which further damaged surrounding tissues and neurons (Gyoneva and Ransohoff, 2015). Additionally, secondary inflammation after CNS injury is the body’s reactive inflammation to the injury, which is different from primary neuroinflammation, such as AD, which is caused by the disorder of normal growth and metabolism in cells (Cao et al., 2021).

In the context of post-injury inflammation, the combination of DAMP (HMGB1, Hsp72, HA, ATP) and TLRs drove the complex inflammation network and astrocyte effector events (Struve et al., 2005; Sun et al., 2017; Sun L. et al., 2019; Du et al., 2021; Li et al., 2021b; Michinaga and Koyama, 2021). Cytokines IL-1β, IL-6, TNF-α activated astrocytes by activating the corresponding receptors and downstream signaling pathways (NFκB, MAPK, NO synthase), and led to the secretion of inflammatory substances (HMGB1, NO, ROS) which further promoted the activation cascade of astrocytes (Swanson et al., 2004; Sun et al., 2017; Sun L. et al., 2019; Patil et al., 2021; Qian et al., 2021). Human spinal cord astrocytes induced by IL-1β showed up-regulation of chemokines and axon permissive factors (including FGF2, BDNF, and NGF) expression, and down-regulation of most genes that regulate axon suppression molecules, including ROBO1 and ROBO2 (Teh et al., 2017). After the injury, the EGFR of astrocytes is up-regulated, and mTOR pathway is up-regulated after combining with EGF. The use of EGFR inhibitors effectively reduced reactive astrogliosis (Codeluppi et al., 2009; Li Z. W. et al., 2014). You et al. (2017) proposed that IL-17-JAK/STAT-VEGF axis was involved in the activation of astrocytes after SCI. As a clear target of MIF, the CD74 receptor on the astrocyte membrane binded to MIF, leading to excessive activation of astrocytes, and this process was significantly blocked by c-Jun N-terminal kinase inhibitors (Zhou et al., 2018). But in gecko astrocytes, the combination of MIF and CD74 could not cause obvious inflammation. Du et al. (2021) proved that Vav1 was the key mediator of this phenomenon. In addition, lncRNAPVT1/miR-186–5p/CXCL13/CXCR5 axis and lncRNA H19/miR-1–3p/CCL2 axis were involved in the activation of astrocytes after SCI (Li P. et al., 2020; Zhang P. et al., 2021). MiR-21 regulated the proliferation, secretion, and activation of astrocytes through the PI3K/Akt/mTOR signaling pathway mediated by PTEN, as a positive factor for the recovery of acute SCI (Liu et al., 2018). MiR-17–5p may specifically regulate the proliferation of RAs triggered by LIF through the JAK/STAT3 pathway (Hong et al., 2014). miR-379 (A et al., 2019), miR-124 (Jiang et al., 2020), miR-145 (Wang et al., 2015), and miR-140 (Tu et al., 2017) negatively regulated astrocyte activation and improved the prognosis of the disease. The transcription factors OLIG2 and SP1, as well as FGF, FGFR, and PDGFRβ have all been implicated in glial scar formation (Kang et al., 2014; Koyama, 2014; Pei et al., 2017; Table 1). These experimental results obtained in ideal places under different conditions emphasized the heterogeneity of reactive astrocytes at the morphological, functional, biochemical, metabolic, and transcriptome levels. In the complex environment inside the body, they will be covered up.

**Table 1:** Molecules and signaling pathways that involved in the activation of astrocytes.

Etiology category	Activation factor
Primary mechanical force	Plasma membrane stretching (Neary et al., 2003, 2005), Cav1.2 voltage-gated Ca^2+^ channels (Cheli et al., 2016; Zamora et al., 2020), high-rate overpressure (Hlavac and VandeVord, 2019; Hlavac et al., 2020), fluid shear stress (Wakida et al., 2020).
Cytokines and growth factors	IL-1β (Teh et al., 2017), IL-6 (Patil et al., 2021), IFN-γ, CNTF, EGF (Li Z. W. et al., 2014), IL-17 (You et al., 2017), TNF-α (Gayen et al., 2020; Patil et al., 2021), LIF (Kerr and Patterson, 2004; Goodus et al., 2016), VEGF (Gao et al., 2015), MIF (Du et al., 2021), FGF (Kang et al., 2014), CTGF (Lu M. et al., 2019).
Chemokines	MCP-1 (Gwak et al., 2012; Joy et al., 2019; Liraz-Zaltsman et al., 2021).
Signal transducers	STAT3, NF-κB, JAK2 (Oliva et al., 2012; You et al., 2017; Li X. et al., 2020), mTOR (Codeluppi et al., 2009), Notch1 (Ribeiro et al., 2021), MAPK (Zhang et al., 2021), ERK (Sticozzi et al., 2013; Li et al., 2021a), PKC (Chao et al., 2018), SOX9 (Liu W. et al., 2021).
Receptors	p75NTR (Chen et al., 2020), CB2R (Jing et al., 2020), ET_B_R (Koyama, 2021), EGFR (Li Z. W. et al., 2014), TLRs (Kigerl et al., 2014; Rosciszewski et al., 2018), purine receptor (Li et al., 2021b), FGFR (Kang et al., 2014), PDGFRβ (Pei et al., 2017), CD36 (Bao et al., 2012), CD44 (Bourguignon et al., 2007), CD74 (Su et al., 2017).
Chaperone proteins	Sig-1R, Hsp72, PDIs (Michinaga and Koyama, 2021).
Hormones	Neuron-derived estrogen (Lu Y. et al., 2020), noradrenalin (Smith et al., 2005; Bekar et al., 2008).
Oxidative stress molecules	NO (Swanson et al., 2004), ROS (Qian et al., 2021).
Non-coding RNA	lncRNAPVT1/miR-186–5p (Zhang P. et al., 2021), lncRNA H19/miR-1–3p (Li P. et al., 2020), miR-21 (Liu et al., 2018), miR-145 (Wang et al., 2015), miR-140 (Tu et al., 2017), miR-17 (Hong et al., 2014), miR-379 (A et al., 2019), miR-124 (Jiang et al., 2020).
Transcription factor	Olig2, Sp1 (Koyama, 2014).
Protease	uPA (Diaz et al., 2021), USP18 (Liu W. et al., 2021).
Proteins	HMGB1 (Sun et al., 2017; Sun L. et al., 2019), ICAM-1 (Gwak et al., 2012), Galectin-3 (Ribeiro et al., 2021).
Peptides	ET-1 (Goodwin and Grizzle, 1994; Michinaga et al., 2018, 2020a).
Others	HA (Struve et al., 2005), Glutamate (Gwak et al., 2012), ATP, Ca^2+^ (Li et al., 2021b), NG_2_ (Huang et al., 2016), Cr (Ma et al., 2017).

## Reactive Astrocytes

RAs are astrocytes that undergo molecular, morphological, and functional changes in response to pathological stimuli from surrounding tissue, such as CNS disease, injury, and deleterious experimental manipulation, among others. As mentioned before, the lifelong adaptive plasticity of astrocytes and the complexity of the disease background determine the diversity of astrocyte subpopulations after injury. In animal models of TBI, P2Y (1)R stimulation was shown to reduce the severity of brain edema and cytotoxic swelling (Talley Watts et al., 2013). However, the results of another study suggested that microglia could convert astrocytes into neurons by mediating the downregulation of P2Y (1)R (Shinozaki et al., 2017). Early et al. (2020) proposed that astrocytes exhibited age-related progressive reactive astrocyte response by the models of TBI in mice of different ages. Recently, Hasel et al. (2021) successfully demonstrated the heterogeneity of RAs in the brain of LPS-induced mouse models. They used single-cell sequencing combined with spatial transcriptomics and *in situ* hybridization techniques to show that RAs were transcriptome and spatially heterogeneous under inflammatory conditions; and clarified the highly expressed genes and possible functions of RA subtypes in different anatomical locations (Hasel et al., 2021). Combined, the findings of all these studies have highlighted the high heterogeneity of RAs, which can lead to both neuroprotective and toxic effects after CNS injury (Miller, 2018). Differences in *in vitro* induction conditions; species used in animal models; injury type, degree, and location; and time passed after the injury have all contributed to the contrasting results obtained in different studies. All these make the precise typing of RAs more difficult.

### Debris Clearance

The timely removal of dead cells after CNS injury helps limit secondary tissue damage. Phagocytosis is normally carried out by professional phagocytes. However, several electron microscopy-based studies as early as the 1970s showed that astrocytes could swallow small fragments, such as axons or myelin fragments (Ronnevi, 1978). Later, it was discovered that astrocytes were involved in the removal of myelin debris during Wallerian degeneration in the goldfish visual system (Colavincenzo and Levine, 2000). Subsequent studies showed that after CNS injury, astrocytes participate in the removal of axons and myelin fragments, even entire dead cells, thereby protecting injured neurons from contact-induced cell death (Basiri and Doucette, 2010; Lööv et al., 2012). Morizawa et al. (2017) reported that following brain ischemia, RAs could become phagocytic in a limited spatiotemporal pattern and engulf debris *via* upregulating the phagocytosis-related ABCA1 pathway. Wang et al. showed that astrocytes directly cleared myelin debris through endocytosis after SCI (Wang S. et al., 2020).

### Glutamate Excitotoxicity

A sharp increase in extracellular glutamate levels has been detected in both CNS injury models and human patients, and this increase represents the cumulative effect of several pathological events that lead to the overstimulation of glutamate receptors and the occurrence of large cation fluxes (Lima et al., 2021). Glutamate excitotoxicity plays an important role in the development of secondary CNS injury. It can lead to neuronal death, followed by prolonged depolarization and subsequent ion imbalance, ATP depletion, increased intracellular free calcium levels, and, ultimately, more serious tissue damage (Jamjoom et al., 2021).

The glutamate transporters GLAST and GLT-1 are mainly expressed in astrocytes and are downregulated following TBI, which leads to enhanced excitotoxicity (Beitchman et al., 2020). Astrocytic excitatory amino acid transporters (EAATs) can protect against neuronal death induced by microglia-derived glutamate, whereas microglial EAATs exert neither neurotoxic nor neuroprotective effects (Liang et al., 2008). These observations indicate that astrocytic glutamate transporters are key for limiting the development of excitotoxic conditions by reducing the concentration of interstitial glutamate. *In vitro*, oxygen–glucose deprivation/reoxygenation insult can reportedly activate the HMGB1/TLR4 axis and reduce glutamate clearance by inhibiting GLAST expression in primary astrocytes (Lin et al., 2020). Similarly, the downregulation of GLT-1 expression in RAs leads to worse functional and histological outcomes following SCI (Lepore et al., 2011a, b). In addition, during cerebral hemorrhage, astrocytic volume-regulated anion channels release glutamate, further aggravating the damage (Yang J. et al., 2019). Interestingly, Li et al. illustrated that the overexpression of the astrocytic glutamate transporter GLT1 exacerbated phrenic motor neuron degeneration, diaphragm impairment, and forelimb motor dysfunction post cervical contusion SCI, while the transplantation of glial progenitors that overexpress the glutamate transporter GLT1 could overcome the diaphragm dysfunction (Li K. et al., 2014; Li et al., 2015).

### Cytotoxic Edema

After CNS injury, the brain and spinal cord tissues undergo edema, leading to intracranial or medullary cavity hypertension, secondary to more serious tissue damage that may lead to fatal brain injury or hernia (Liang et al., 2007). Many studies have shown that the degree of cerebral and spinal cord edema is associated with the severity of trauma and subsequent motor dysfunctions (Miyanji et al., 2007). Cytotoxic edema is characterized by the swelling of all cell types due to excessive water retention. In contrast, astrocytes are the main cause of brain swelling in brain edema (Liang et al., 2007). AQP-4, expressed in the brain (perivascular and subpial membrane domain) and spinal cord astrocytes, is the most abundant aquaporin in the CNS and represents a major pathway for the entry of excess water into damaged tissue (Nesic et al., 2006; Tait et al., 2008; Saadoun and Papadopoulos, 2010). Astrocytic AQP-4 is primarily responsible for cytotoxic edema after CNS injury (Amiry-Moghaddam et al., 2003).

**Figure 2:**
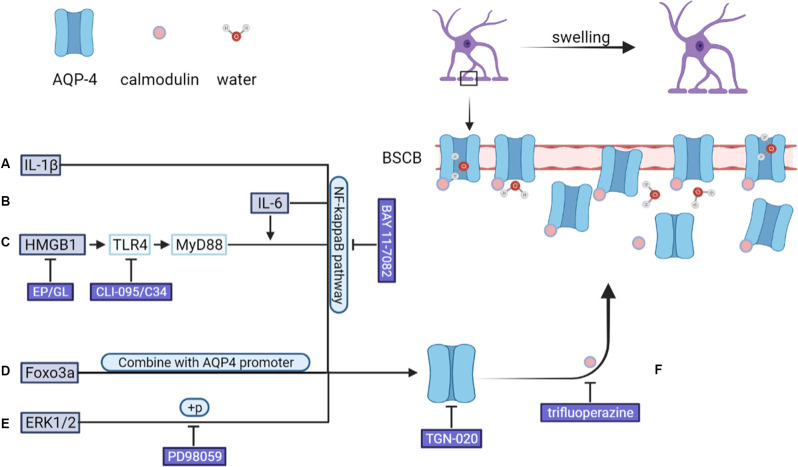
After CNS injury, an increase in the levels of **(A)** IL-1β and **(B)** IL-6 leads to the upregulation of AQP-4 expression through the NF-κB pathway. **(C)** HMGB1 upregulates AQP-4 expression *via* the HMGB1/TLR4/MyD88/NF-κB axis independently of IL-6. **(D)** FOXO3A undergoes nuclear translocation, binds to the *AQP4* promoter, and upregulates AQP-4 expression. **(E)** SCI-induced upregulation on of AQP-4 expression was down-regulated by PD98059 (ERK blocking agent) and TGN-020 (aquaporin-4, AQP4, blocking agent). In addition, **(F)** AQP-4 undergoes a conformational change after binding to calmodulin, after which it localizes to the BSCB, leading to an increase in the amount of water entering astrocytes. ERK, extracellular signal-regulated protein kinase; BSCB, blood–spinal cord barrier.

In animal models of CNS injury, AQP-4 mRNA and protein expression levels are significantly upregulated in activated astrocytes (Finnie et al., 2011; Hemley et al., 2013). Various mechanisms are involved in this process in astrocytes, such as IL-6/NF-κB pathway activation, HMGB1/TLR4/MyD88/NF-κB signaling pathway activation, FOXO3A nuclear translocation, and ERK1/2 phosphorylation (Ito et al., 2006; Kapoor et al., 2013; Sun et al., 2017; Sun L. et al., 2019; Zhang et al., 2019a; Li et al., 2021a). Experiments conducted using AQP-4-deficient mice showed that AQP-4 promotes the formation of cytotoxic edema, whereas the absence of AQP-4 reduces edema severity after acute water intoxication, ischemic stroke, and SCI (Manley et al., 2000; Saadoun et al., 2008). In the rat model of TBI, AQP-4 knockdown reportedly reduces the extent of cytotoxic and post-traumatic brain edema (Lu H. et al., 2020). Kitchen et al. suggested that brain or spinal cord swelling was not only related to the total expression of AQP-4, but also the subcellular translocation of AQP-4 to the BSCB. Their data showed that calmodulin could directly bind to the carboxyl terminus of AQP-4, resulting in specific conformational changes and AQP-4 cell-surface localization. In rat SCI models, trifluoperazine-mediated calmodulin inhibition suppressed AQP-4 localization to the BSCB, led to the ablation of CNS edema, and resulted in accelerated functional recovery relative to that seen in untreated animals (Kitchen et al., 2020). As shown in Figure 2. As AQP-4 cell surface localization is controlled by calcium/protein kinase A/calmodulin in astrocytes, targeting calmodulin may also represent a novel treatment method for cytotoxic edema (Kitchen et al., 2015, 2020). In addition to AQP-4, other functional molecules in astrocytes, such as NKCC1, Sur1/Trpm4, AQP-1, and vasopressin are also considered to be initiators of cytotoxic edema formation (Nesic et al., 2008; Jayakumar et al., 2011; Jia et al., 2016; Gerzanich et al., 2019).

### BBB/BSCB: Disruption or Recovery

CNS damage can lead to the loss of BBB/BSCB integrity. Astrocytes regulate BBB/BSCB homeostasis through end-feet processes that surround endothelial cells. A series of factors derived from RAs after an injury have opposing effects on the BBB/BSCB (Michinaga and Koyama, 2019; Table 2).

**Table 2:** Factors destroy or recover BBB/BSCB.

BBB/BSCB destruction	BBB/BSCB recovery
NO (Sharma et al., 2005, 2019; Saha and Pahan, 2006; Buskila et al., 2007; Gu et al., 2012; Jiang et al., 2014)	MANF (Li et al., 2018)
Excess glutamate (András et al., 2007; Liu et al., 2010; Sulejczak et al., 2016; Lu L. et al., 2019)	Shh (Xia et al., 2013; Xing et al., 2020; Yue et al., 2020; Michinaga et al., 2021)
VEGF (Gao et al., 2015; You et al., 2017)	Ang-1 (Xia et al., 2013; Sabirzhanov et al., 2019; Sun J. D. et al., 2019; Michinaga et al., 2020b)
MMP-9 (Noble et al., 2002; Michinaga et al., 2018; Liu et al., 2020)	fatty acid-binding protein 7 (Rui et al., 2019)
ET-1 (Michinaga et al., 2018; Michinaga et al., 2020a; Michinaga et al., 2021)	RA (Mizee et al., 2014; Kong et al., 2015; Zhou et al., 2016)
APOE4 variant (Main et al., 2018; Montagne et al., 2020)	IGF-1 (Bake et al., 2016, 2019; Pitt et al., 2017; Li H. et al., 2020)
	APOE4 (Main et al., 2018)

Nitric oxide (NO) and excess glutamate derived from RAs after an injury can damage the BBB and the BSCB (Saha and Pahan, 2006; András et al., 2007; Lu L. et al., 2019; Sharma et al., 2019). In animal models of TBI and SCI, the expression of VEGF and MMP-9, both factors that promote BBB permeability, increases in RAs, and inhibiting them reduces BBB/BSCB-related damage after injury (Noble et al., 2002; Gao et al., 2015; You et al., 2017; Michinaga et al., 2018; Liu et al., 2020). Astrocyte-derived ET-1 was shown to induce the upregulation of ICAM-1 and VCAM-1 expression in human brain microvascular endothelial cells and aggravate the destruction of the BBB. ET receptor antagonists such as bosentan, BQ788, and S-0139 can alleviate the loss of BBB integrity in TBI model mice (McCarron et al., 1993; Matsuo et al., 2001; Michinaga et al., 2018, 2020a). Interestingly, studies on mice have highlighted that the APOE E4 variant (APOE4) is a risk factor for poor outcomes in CCI. However, APOE is an important modulator of spontaneous BBB stabilization following TBI (Main et al., 2018; Montagne et al., 2020). Astrocyte-derived neurotrophic factor (MANF) can inhibit inflammation and promote angiogenesis and BBB repair (Li et al., 2018). Astrocyte ablation results in the failure of BSCB repair, local tissue destruction, severe demyelination, and the death of neurons and oligodendrocytes following SCI (Faulkner et al., 2004). After CNS injury, the expression of Shh is increased in astrocytes. The administration of exogenous Shh attenuates BBB destruction, while the application of the Shh inhibitor jervine exerts the opposite effects in mice with TBI (Xing et al., 2020; Michinaga et al., 2021). In the mouse SCI model, Shh/Gli1 signaling is induced in RAs and plays an important role in the permeability of BSCB and locomotor recovery after SCI (Yue et al., 2020). The expression of ANG-1 in astrocytes is decreased after CNS injury, while the administration of recombinant ANG-1 can alleviate the destruction of the BBB/BSCB (Sabirzhanov et al., 2019; Michinaga et al., 2020b). Astrocyte-derived FABP7 enhances BBB integrity through the caveolin-1/MMP signaling pathway after TBI, and displays neuroprotective properties after SCI (Rui et al., 2019; Senbokuya et al., 2019). In addition, astrocyte-derived retinoic acid and IGF-1 have also been shown to participate in BBB/BSCB maintenance and vascular protection (Kong et al., 2015; Bake et al., 2016; Zhou et al., 2016; Li H. et al., 2020). Notably, Shh and MMP-9 can restore or disrupt the BBB or BSCB through multiple mechanisms, and both proteins have the potential to serve as therapeutic targets for CNS injury.

### Inflammation: Basic Protective Function and the Consequences of Overactivation

Inflammation represents a physiological protective response to injury; however, extreme inflammation, which is inevitable following CNS injury, results in additional tissue damage (Popovich and Jones, 2003; Förstner et al., 2018). RAs promote inflammation after CNS injury by secreting cytokines, chemokines, reactive oxygen species (ROS), NO, and damage-associated molecular patterns, all factors that are involved in the activation of microglia and the recruitment of peripheral immune cells, thereby maintaining and even further aggravating neuroinflammation (Wicher et al., 2017; Linnerbauer et al., 2020). The NF-kB signaling pathway in RAs is a key regulator of inflammation in the CNS (O’Neill and Kaltschmidt, 1997). In animal models of CNS injury, NF-κB is highly activated and the expression of NF-kB-dependent genes is upregulated (Schneider et al., 1999). Inhibiting NF-κB signaling dampens astrocyte responses to brain injury, resulting in neuroprotective effects (Acarin et al., 2001; Brambilla et al., 2005). An *in vitro* study showed that ATP-stimulated human astrocytes activated NLRP2 inflammasomes, while the knockdown of NLRP2 significantly reduced the inflammatory response in human astrocytes (Minkiewicz et al., 2013). Many other pro-inflammatory molecules have been associated with astrocyte reactivity, such asS100β, ICAM-1, PrPc, TrkB, D-dopachrome tautomerase, and MIF (Kabadi et al., 2015; Zhang et al., 2019b; Charkviani et al., 2020; Ji et al., 2021; Sulimai et al., 2021). However, using a mouse model of TBI, Myer et al showed that RA ablation aggravated cortical degeneration after moderate CCI, but did not affect cortical degeneration following severe CCI, which suggested that RAs also have a basic protective role in inflammation after injury (Myer et al., 2006). Similar results were obtained with astrocyte ablation after SCI (Faulkner et al., 2004). Long et al. (2020) showed that astrocyte-derived exosomes enriched with miR-873a-5p can inhibit the NF-κB signaling pathway and promote the transformation of protective M2 microglia, thereby inhibiting excessive neuroinflammation. Additionally, Zaheer et al. (2001) showed that activation of the NF-κB signaling pathway resulted in the synthesis of neurotrophic factors (nerve growth factor and brain-derived neurotrophic factor), which is essential for neuronal survival after injury.

## RA Subgroup with Neurotoxicity

As early as 2012, Zamanian et al. (2012) discovered a potentially harmful subgroup of RAs. Subsequently, Liddelow et al. (2017) proposed a neurotoxic RA with C3 as a molecular marker and named it A1 astrocytes. A1 astrocytes were induced by cytokines (TNF-α, IL-1α, and complement component C1q) secreted by activated microglia. Although the concept of A1 is not relevant in this field, many previous research results of A1 neurotoxic astrocytes can help subsequent research on the neurotoxic subpopulations of RAs. A1 astrocytes lose many basic functions and gain harmful ones when compared with normal astrocytes. Namely, A1 astrocytes have fewer synapses and a weaker ability for synapse induction; impaired myelin scavenging ability; they can inhibit oligodendrocyte maturation; exhibit stronger neurotoxicity; and kill CNS neurons that have severed axons (Liddelow et al., 2017; Li X. et al., 2020). A1 astrocytes have a significantly different morphology: long dendrites (Zou et al., 2019). This suggests that the morphology of RAs may be changeable. Adding morphological features to the subgroup division can make the typing more specific and accurate. A1 astrocytes are found in a variety of CNS injuries and neurodegenerative diseases but are also present during the normal aging process (Clarke et al., 2018; Yun et al., 2018; Zheng et al., 2021). Alawieh et al. showed that a significant increase in C3 levels after CNS injury triggers continuous microglia degeneration and astrocyte activation, reduces dendrite and synapse density, and ultimately leads to the loss of neurons (Alawieh et al., 2018; Clark et al., 2019). After SCI, mice with C3 deficiency have reduced inflammation and secondary damage and better nerve regeneration and functional recovery after injury compared with that for normal mice (Guo et al., 2010). However, mice with C3aR deficiency show abnormal neurodevelopment that persists into adulthood, and is characterized by locomotive hyperactivity and altered cognitive functions (Pozo-Rodrigálvarez et al., 2021). Wang et al. (2021) proposed a more radical possibility, namely, that A1 astrocytes could directly kill neurons by secreting neurotoxic C3. Several studies have reported that C3 is closely related to the onset of multiple neurodegenerative diseases (Lian et al., 2015; Litvinchuk et al., 2018). These observations suggest that the basic C3 level is necessary for the maintenance of a normal physiological environment in the CNS, whereas excessive C3 availability produces neurotoxic effects after injury. However, it must be acknowledged that the expression of a singular marker “C3” is not a definitive marker that identifies A1 astrocytes. The work of Boisvert et al. (2018) showed that C3 was upregulated on astrocytes in the condition of aging, and did not necessarily, or categorically, indicate A1 astrocytes. Therefore, it is neither accurate nor objective that C3 is used as a singular marker of A1 astrocytes in injury and diseases in humans and other models. Recently, Guttenplan et al. (2021) proposed that saturated lipids contained in APOE and APOJ lipid particles mediated the neurotoxicity of RAs. Astrocytes specifically knock out saturated lipid synthase ELOVL1 to eliminate the formation of long-chain saturated lipids, which reduced astrocyte-mediated toxicity.

In CNS injury, a variety of substances and intracellular signal pathways are involved in the induction and transformation of the functions of RAs (neurotoxicity and neuroprotection; Table 3). For instance, the activation of the NF-κB and Notch signal pathways promotes A1 transformation, while exposure to mesenchymal stem cell (MSC)-derived exosomes, which play anti-inflammatory and neuroprotective roles after SCI, suppresses A1 astrocyte numbers by inhibiting the NF-κB signaling pathway (Wang et al., 2018; Liu et al., 2019; Qian et al., 2019). Additionally, activating the FGF2/FGFR1 pathway can reverse the increase in C3 expression levels in astrocytes following ultrasound exposure (Zou et al., 2019). After SCI, the application of electrospun fiber was reported to promote the expression of A1-specific markers, but electrospun fiber-containing TGF elicited the opposite effect (Gottipati et al., 2020). In comparison, in an IL-1β-induced neonatal rat model of white matter injury, astrocytes showed A2 reactivity (Shiow et al., 2017). After TBI, neuron-derived prokineticin 2 and astrocyte-derived estrogen activated STAT3 signaling pathway in astrocytes, leading to the upregulation of A2 astrocytes (Neal et al., 2018; Ma et al., 2020; Wang J. et al., 2020). We have previously shown that miR-21, a regulator of the STAT3 pathway, can transform neurotoxic (A1) RAs into an A2 phenotype (Su et al., 2019). MFG-E8, MSC-derived extracellular vesicles (EVs), Wnt-3a, and Trkβ have also been shown to be involved in A1/A2 transformation (Xu et al., 2018; Zhang D. et al., 2019; Kaminski et al., 2020; Miyamoto et al., 2020). Interestingly, FGF2 can inhibit the TGF-β1-induced increase in GFAP expression in astrocytes (Tran et al., 2018). The antagonism between different molecules that induce the same phenotype further underlines the need for the development of a more precise method for typing RAs.

**Table 3:** Neurotoxic astrocyte-related substances and signal pathways.

Effect	Inductive molecule	Signal path	Reference
Reduce neurotoxicity	MSC-exo	NF-κ (-)	Wang et al. (2018) and Liu et al. (2019)
	HSF1	NF-κB (-) MAPKs (-)	Li L. et al. (2021)
	–	Notch (-)	Qian et al. (2019)
	–	FGF2/FGFR1 (+)	Zou et al. (2019)
	TGF-β3	–	Gottipati et al. (2020)
Induce neuroprotection	IL-1β	–	Shiow et al. (2017)
	Astrocyte-Derived Estrogen	JAK-STAT3 (+)	Wang J. et al. (2020)
	PK2	STAT3 (+)	Neal et al. (2018) and Ma et al. (2020)
Reduce neurotoxicity and Induce neuroprotection	miR-21	STAT3 (+)	Su et al. (2019)
	MFG-E8	PI3K-Akt (+) & NF-κB (-)	Xu et al. (2018)
	MSC-EVs	–	Kaminski et al. (2020)
	Wnt-3a	Wnt/β-catenin signaling pathway (+)	Zhang D. et al. (2019)
	Trkβ	–	Miyamoto et al. (2020)

## Glial Scars and SAS

Following CNS injury, naive astrocytes transform into RAs, and then eventually SAs, leading to impaired axon regeneration and functional recovery. This continuous phenotypic change is a manifestation of astrocyte reactivity, which was once considered to be a unidirectional and irreversible process (Hara et al., 2017). Diseases and injuries of the CNS are usually accompanied by a certain degree of scar formation, although scar formation differs according to disease and injury (Smith et al., 2015). Glial scars are mainly involved in the repair process after CNS injury. After SCI, damage repair efficiency is low and the resulting pathological changes cannot be overcome. Consequently, here, we focus on astrocyte-mediated scar formation after SCI (Bradbury and Burnside, 2019). SCI lesions exhibit three compartments: a non-neural (stromal) lesion core, astrocyte scar borders, and spared but reactive neural tissue. SAs participate in the formation of astrocyte scar borders (Sofroniew, 2018). The scarring process begins on day 7 post-injury and involves the misalignment of activated astrocytes and the deposition of inhibitory CSPGs. SAs can be identified from 14 dpi (Hara et al., 2017).

Various mediators are involved in glial scar formation, including TGF-β1/2, IFN-γ, FGF, MMP-9, fibrinogen, and STAT3 (Moon and Fawcett, 2001; Herrmann et al., 2008; Hsu et al., 2008; Schachtrup et al., 2010). The glial scar represents a physical barrier that enwraps damaged tissues and restricts the migration of inflammatory cells from the non-neural lesion core to the CNS parenchyma (Voskuhl et al., 2009; Sofroniew, 2015). Glial scars fill the interstitial spaces and induce the formation of new capillaries (Rolls et al., 2009). RA ablation impairs glial scar formation, leading to extensive infiltration of inflammatory cells and loss of neurons (Gu et al., 2019). Importantly, however, RA ablation also exerts an unwelcome inhibitory effect on axon regeneration (Anderson et al., 2016). CSPGs deposited in glial scars inhibit oligodendrocyte precursor cell differentiation and remyelination, the two most important processes underlying axon regeneration. CSPG inhibition or inactivation effectively improves motor function (Bradbury et al., 2002; Silver and Miller, 2004; Siebert et al., 2011; Lang et al., 2015; Tran et al., 2018). Wallerian degeneration of damaged axon protrusions leads to continuous extracellular deposition of axons and myelin debris. Myelin-related molecules (MAG, Nogo, OMGP), in conjunction with CSPGs, inhibit neuronal regeneration and neural plasticity (Sofroniew, 2018). However, the deletion of CSPG-related genes or CSPG receptor blockade only enhances synaptic remodeling and cannot directly overcome the protective effects of the astrocyte scar and lesion cores of non-neural tissue to produce meaningful spontaneous axonal regeneration (Hossain-Ibrahim et al., 2007; García-Alías et al., 2009). A combination of TGF-β1/2 antibodies reduced CNS scar formation in an adult rat model of brain injury; however, this was not accompanied by an increase in axon regeneration (Moon and Fawcett, 2001). GFAP^−/−^vim^−/−^ mice show normal scar formation after TBI or SCI, but the scar density is low and accompanied by bleeding (Pekny et al., 1999). Three genetically targeted loss-of-function interventions—preventing astrocyte scar formation, attenuating scar-forming astrocytes, and ablating chronic astrocytic scars—all failed to promote spontaneous axon regrowth. However, exogenous administration of axon-specific growth factors, coupled with growth-activating priming injuries, stimulated axon regeneration, which was reversed by glial scar ablation (Anderson et al., 2016).

Glial scars transform into fibrous scars 14 dpi, and SAs are produced at the same time. SAs are known to originate from the interaction between RAs and type I collagen *via* the integrin/N-cadherin pathway. Antibodies targeting collagen-binding integrin and N-cadherin neutralizing antibodies both inhibited this process (Hara et al., 2017). Immunofluorescence analysis identified the presence of SOX9-positive nuclei in astrocytes of a wild-type brain scar 30 days after the cortical puncture. In contrast, SOX9 expression was strictly limited to the cytoplasm in the DBN−/− brain. DBN may also participate in the transformation of RAs into SAs (Schiweck et al., 2021). Inhibiting the RA/SA conversion may represent an ideal treatment for CNS injury. For this, the restrictive effect of RAs on inflammation should not be affected, only the formation of the glial scar boundary should be inhibited so as to alleviate the inhibitory effect of the surrounding environment on axon regeneration.

In summary, the dual role of the glial scar in axon regeneration may result from the low inherent regeneration potential of neurons. The growth-activating effect of the glial scar cannot bridge the gap between the neuronal regeneration potential and the physical hindrance represented by glial scars; when a glial scar is ablated, neurons cannot regenerate axons on their own without the growth-activating effect of the glial scar. Han et al. (2020) proposed to increase the intrinsic regenerative power of neurons by restoring cellular energy, and successfully promoted the germination and regeneration of axons after SCI by enhancing mitochondrial transport and energy metabolism. Therefore, in the case of preserving glial scars, enhancing the regeneration potential of neurons may also be a feasible treatment option.

## Strategies for Astrocyte-Targeted Therapy

Based on the dual role of astrocytes in CNS injury, multiple attempts have been undertaken to enhance the beneficial effects of astrocytes or reduce their harmful effects. Here, we mainly review the existing attempts at astrocyte-targeted therapy (Table 4).

**Table 4:** Diverse astrocyte targeted therapy strategies.

Target	Treatment	Model	Mechanism	Curative effect	Reference
Inhibit excessive activation of astrocytes	MP	*In vivo* *In vitro*	Down-regulate astrocyte activation and inhibit CSPG expression	Improve neuron repair and promote neurite outgrowth after excitotoxic injury	Liu et al. (2008)
	Melatonin	*In vivo*	Inhibit astrocyte activation	Reduce neuronal apoptosis	Babaee et al. (2015)
	PPR	*In vivo*	Down-regulate TNF-α, IL-1β, reduce GFAP+ astrocyte cells	Reduce the degree of cerebral edema and seizures	Song Y. et al. (2020)
	TBHQ	*In vivo*	Reduce the production of M1 microglia and inflammatory cytokines, significantly reduce the excessive activation of astrocytes	Reduce neuronal death and lesion volume, improve motor function and cognitive deficits	Zhang et al. (2020)
	AS-IV	*In vitro*	AS-IV reduces the activation of the CXCR4/JNK pathway and ultimately up-regulates the Keap1-Nrf2 signaling	Prevent OGD/R-induced astrocyte apoptosis	Yang J. et al. (2021)
	Simvastatin	*In vivo* *In vitro*	Simvastatin manipulates the caveolin-1 expression in lipid rafts in the astrocyte cell membrane, reduces EGFR phosphorylation, and finally reduces IL-1 production and astrocyte activation	Protect neurons	Li et al. (2009) and Wu et al. (2010)
	ONO-2506	*In vivo*	Inhibit the production of S100B by astrocytes to inhibit the activation of astrocytes	Reduce neuropathic pain after SCI	Ishiguro et al. (2019)
	Edaravone	*In vivo*	Reduce astrocyte proliferation in a rat model of propofol-induced brain injury through the BDNF/TrkB pathway.	Reduce inflammation	Yang Y. et al. (2021)
Reduce Edema	Functionalized Phenylbenzamides	*In vivo* *In vitro*	Reduce AQP-4-mediated water Permeability	Reduce brain edema and improve prognosis	Farr et al. (2019)
	TGN-020	*In vivo*	Inhibit the expression of AQP-4, GFAP, PCNA	Reduce spinal cord edema and promote axon regeneration	Li et al. (2019)
	Atorvastatin	*In vivo*	Inhibit p38MAPK-dependent pathway to down-regulate the expression of AQP4	Reduce ischemic brain edema	Cheng et al. (2018)
	Goreisan	*In vivo*	Decrease AQP-4expression level	Reduce brain water content, alleviate motor deficits	Nakano et al. (2018)
	Trifluoperazine	*In vivo* *In vitro*	Prevent calmodulin from directly binding to the carboxyl terminus of AQP-4, which inhibit AQP-4 localization BSCB	Relieve CNS edema and accelerate functional recovery	Kitchen et al. (2020)
	Bosentan	*In vivo* *In vitro*	Decrease the expression levels of MMP-9, VEGF-A, and Ang-1 in the brain after injury	Reduce BBB dysfunction and cerebral edema	Michinaga et al. (2020a)
	BQ788	*In vivo*	Reduce GFAP-positive astrocytes and their products: VEGF-A and MMP9	Promote the recovery of BBB function and reduce cerebral edema	Michinaga et al. (2018)
	Ulinastatin	*In vivo*	Decrease the activation of ET-1 and inhibit the expression of pro-inflammatory VEGF and MMP-9	Reduce brain edema after TBI	Liu T. et al. (2021)
	EP/GL	*In vivo*	Inhibit the activation of astrocytes, reduce the expression of AQP4, and inhibit the activation of the TLR4/NF-κB signaling pathway	Improve motor function and reduce early spinal cord edema	Sun et al. (2017) and Sun L. et al. (2019)
Astrocyte reprogramming	OCT4, NANOG	*In vitro*		Astrocytes are reprogrammed into the generation of cells expressing neural stem/precursor markers	Corti et al. (2012)
	SOX2	*In vivo*		Resident astrocytes are reprogrammed into proliferating neuroblasts	Niu et al. (2013)
	Zfp521	*In vivo* *In vitro*		Astrocytes are reprogrammed into iNSCs or neurons	Su et al. (2014) and Zarei-Kheirabadi et al. (2019a,b)
	Transcription factors PAX6, NGN2 and ASCL1	*In vitro*		Reprogramming of astrocytes into neurons	Heins et al. (2002) and Berninger et al. (2007)
	Combination of transcription factors Brn-2a, MyT1L, and ASCL1	*In vivo*		Reprogramming of astrocytes into neurons	Torper et al. (2013)
	Transcription factors NeuroD1	*In vivo*		Reprogramming of astrocytes into neurons	Puls et al. (2020)
Reduce the toxicity of RAs and protect neurons	Drug-Loaded Nano-Structured Gel	*In vivo* *In vitro*	Down-regulate A1 astrocytes, reduce iNOS and Lcn2	Improve early exercise ability of injury and protect neurons	Vismara et al. (2020)
	Ponesimod	*In vivo* *In vitro*	Reduce A1 astrocyte polarization by activating the STAT3 signaling pathway	Prevent neuronal death from early brain injury after subarachnoid hemorrhage	Zhang L. et al. (2021)
	Epidermal Growth Factor Hydrogels	*In vitro*	Down-regulate negative A1-like genes (Fbln5 and Rt1-S3) and up-regulate potentially beneficial A2-like genes (Clcf1, Tgm1, and Ptgs2)	Enhance neuroprotection and neuroplasticity	Chan et al. (2019)
	RTMS	*In vivo* *In vitro*	Reduce the production of inflammatory mediators, promote HIF-1α signaling, transform A2 astrocytes into A1 astrocytes	Reduce neuronal apoptosis, promote blood vessel repair, and improve cognitive function.	Zong et al. (2020)
	Physical exercise	*In vivo*	Down-regulate the expression of IL-1α, C1q, and TNF, up-regulate the release of TGFβ, and promote the conversion of A1astrocytes to A2 astrocytes	Promote white matter repair and cognitive improvement	Jiang et al. (2021)
	RvD1	*In vivo* *In vitro*	Induces higher levels of mitochondrial autophagy in astrocytes to protect the mitochondrial morphology and membrane potential of the astrocytes	Reduce cognitive impairment and brain edema, improve the neuron survival rate after TBI	Ren et al. (2020)
	Baicalin	*In vivo* *In vitro*	Inactivate SDH to inhibit ROS production and reduce the loss of GS protein in astrocytes after injury	Reduce excitotoxicity and protect neurons	Song X. et al. (2020)
	LEC	*In vivo*	Reduce lipid peroxidation of astrocytes and increase their glutamate uptake	Reduce excitotoxicity and protect neurons and oligodendrocytes	Lima et al. (2021)
	Agathisflavone	*In vitro*	Increase the expression of neurotrophic factors, reduce the expression of GFAP and hypertrophy of astrocytes	Protect neurons and promote neurite growth	de Amorim et al. (2020)
	Ganglioside GM1	*In vivo* *In vitro*	GM stimulates the expression of genes related to glucose metabolism and enhances glycolysis in astrocytes	Protect neurons	Finsterwald et al. (2021)
Others	Sodium houttuyfonate	*In vivo* *In vitro*	Reduce NLRP3 inflammasome activation, TLR4 activity, phosphorylation of ERK and NF-κB	Reduce inflammation and promote angiogenesis	Yao et al. (2021)
	Ferrostatin-1	*In vitro*	Suppress the ROS levels and activate the Nrf2/HO-1 signaling pathway	Alleviate astrocytes inflammation and ferroptosis	Li S. et al. (2021)

### Inhibit Excessive Activation of Astrocytes

In the inflammatory phase after CNS injury, excessive activation of astrocytes aggravates the inflammatory cascade and has a negative impact on the prognosis of the disease (Johnson et al., 2013). Methylprednisolone (MP) is a typical representative of an RA-targeting molecule that has already been used in the clinic. MP can reduce astrocyte activation and downregulate the expression of CSPG, thereby promoting the growth of neurites after injury (Liu et al., 2008). Melatonin can exert similar effects (Babaee et al., 2015). PPR, TBHQ, AS-IV, and simvastatin can all reduce the production of inflammatory mediators and inhibit excessive astrocyte activation, thereby protecting neurons and improving prognosis (Li et al., 2009; Wu et al., 2010; Song Y. et al., 2020; Zhang et al., 2020; Yang J. et al., 2021). ONO-2506 can also attenuate astrocyte activation, thus minimizing secondary damage and relieving neuropathic pain after SCI (Ishiguro et al., 2019). As a variety of free radical scavengers, edaravone alleviated astrocyte proliferation and inflammation in a rat model of propofol-induced brain injury (Yang Y. et al., 2021). The selective inhibitor of D-dopachrome tautomerase, a close homolog of MIF protein, effectively attenuated the inflammatory activation of astrocytes after SCI and improves motor function, which helps to develop the application of anti-inflammatory drugs in CNS injuries (Ji et al., 2021). In fact, anti-inflammatory drugs have been used in the clinical treatment of CNS injuries for a long time.

### Reduce Edema

AQP-4 is the best-characterized astrocyte-related molecule. Functionalized phenylbenzamide, TGN-020, atorvastatin, and goreisan all target AQP-4, improving post-injury edema and prognosis (Cheng et al., 2018; Nakano et al., 2018; Farr et al., 2019; Li et al., 2019). Using a rat model of SCI, Kitchen et al administered trifluoperazine to inhibit the direct binding of calmodulin to the carboxyl terminus of AQP-4, which inhibited its localization to the BSCB. This effect relieved CNS edema and accelerated functional recovery relative to untreated animals (Kitchen et al., 2020; Figure 2). However, in a review by Nesic et al. (2010), the authors proposed that the therapeutic effect of AQP-4 depends not only on the time interval after SCI or the animal model but also on the balance between the protective effect of increased AQP-4 levels on hypoxia and the harmful effects associated with sustained astrocyte swelling. ET-1 has also received widespread attention as a putative therapeutic target. Both bosentan (an ET_A_/ET_B_ antagonist) and BQ788 (an ET_B_ antagonist) effectively attenuated BBB disruption and cerebral edema in both patients and mice with TBI, whereas the ET_A_ antagonists ambrisentan and FR139317 elicited no effect (Michinaga et al., 2018, 2020a; Liu T. et al., 2021). This suggests that the deleterious effect of ET-I following CNS injury mainly depends on ET_B_R. Additionally, EP/GL inhibited the activation of astrocytes, reduced the expression of AQP4 and early spinal cord edema (Sun et al., 2017; Sun L. et al., 2019).

### Reduce the Toxicity of RAs and Protect Neurons

A drug-loaded nano-structured gel and ponesimod were shown to improve motor performance in the early stages after injury and protect neurons by suppressing the activation of the neurotoxic phenotype of RAs (Vismara et al., 2020; Zhang L. et al., 2021). Epidermal growth factor-containing hydrogels can reportedly alter astrocyte behavior, i.e., they downregulate the expression of deleterious neurotoxicity-related genes (*Fbln5* and *Rt1-S3*) while upregulating that of potentially beneficial neuroprotective phenotype-associated genes (*Clcf1*, *Tgm1*, and *Ptgs2*), thereby indirectly enhancing neuroprotection and neuroplasticity (Chan et al., 2019). RTMS, HSF1, and physical exercise also lead to the conversion of the neurotoxic phenotype into the neuroprotective phenotype, which promotes functional recovery after injury (Zong et al., 2020; Jiang et al., 2021; Li L. et al., 2021). Mitochondria may also play a role in A1 polarization. Incubation with cobalt chloride (CoCl2) converted astrocytes from an A2 to an A1 state, concomitant with a reduction in mitochondrial migration. Trkβ agonists can convert A1 astrocytes to an A2 phenotype *via* reducing mitochondria migration (Miyamoto et al., 2020). Mitochondrial transplantation after CNS injury decreases the release of inflammatory factors such as IL-1β and TNF-α and significantly suppresses astrocyte and microglia activation, thus protecting neurons and promoting functional recovery (Zhang Z. et al., 2019). Resolvin D1 protected mitochondrial morphology and membrane potential in astrocytes, removed damaged mitochondria and thereby enhanced the survival of neurons (Ren et al., 2020). This prompts us to pay attention to the impact of the energy status of RAs on their function in the context of disease. A better understanding of the changes occurring in mitochondrial morphology and function after CNS insult may yield novel strategies for the treatment of CNS injuries. Baicalin and LEC were shown to stabilize astrocytes after injury and increase their glutamate uptake, effects that can reduce excitotoxicity and protect both neurons and oligodendrocytes (Song X. et al., 2020; Lima et al., 2021). Agathisflavone and ganglioside GM1 promoted the neuroprotective effect of astrocytes (de Amorim et al., 2020; Finsterwald et al., 2021).

### Astrocyte Reprogramming

Astrocytes retain limited neural stem cell potential and can be reprogrammed into a stem cell-like state to replenish neurons lost after injury (Kriegstein and Alvarez-Buylla, 2009; Verkhratsky and Nedergaard, 2018). The transcription factors OCT4, SOX2, NANOG, and zinc-finger nuclear protein Zfp521 can individually reprogram mature astrocytes into neural stem cells (Corti et al., 2012; Niu et al., 2013; Su et al., 2014; Yang H. et al., 2019; Zarei-Kheirabadi et al., 2019b). The transcription factors PAX6, NGN2, and ASCL1, participate in the transformation of astrocytes into neurons *in vitro* (Heins et al., 2002; Berninger et al., 2007), similar to that seen with the combination of three nerve conversion factors (ASCL1, Brn-2a, and MyT1L) *in vivo* (Torper et al., 2013). Noristani et al. (2016) showed that more than 10% of autologous astrocytes were transdifferentiated and expressed classic neural stem cell markers after SCI. Decreased Notch signaling due to stroke was shown to be necessary for astrocyte neurogenesis (Magnusson et al., 2014). The transcription factors NeuroD1, SOX2, and ZFP521 can all be used to reprogram astrocytes into neurons or neural stem cells after SCI (Zarei-Kheirabadi et al., 2019a; Puls et al., 2020).

### Others

Sodium houttuyfonate effectively inhibited the activation of microglia cells while promoting the activation of astrocytes and angiogenesis (Yao et al., 2021). Ferrostatin-1 alleviated astrocytes inflammation and ferroptosis by suppressing the ROS levels and activating the Nrf2/HO-1 signaling pathway (Li S. et al., 2021). Additionally, many other molecules, such as USP18 (Liu W. et al., 2021), p-ERK1/2 (Li et al., 2021a), CREB (Pardo et al., 2016), HSPA12B (Xia et al., 2016), CCR5 (Joy et al., 2019), also represent potential therapeutic targets that merit further investigation.

Although attention has bright prospects, the difficulty in obtaining human CNS tissue and the substantial differences between rodents and human astrocytes (Zhang et al., 2016) represent unavoidable obstacles to the identification or development of strategies for the treatment of CNS injury, that is, how to translate research results from animal studies to humans. Although astrocytes induced by human pluripotent stem cells provide a possible cell model, these astrocytes differ from astrocytes under normal physiological conditions, at least partially. How to transfer research results from animal models to human patients will likely also be the focus of research attention in the future.

## Conclusions

The importance of astrocytes in CNS disease and injury is widely recognized; however, our understanding of astrocyte functions is still in its infancy. The continuous development and breakthrough of instruments and technologies provide conditions for accurate typing of astrocytes. The combination of single-cell and spatial transcriptome sequencing shows promise as a means of determining astrocyte heterogeneity after injury. Through the sequencing of several key times after injury, the time and space distribution of each astrocyte subpopulation can be determined. For example, astrocyte subpopulation D appears on the 7th day after SCI, mainly distributed in the core of injury. Further investigations to determine the temporal and spatial specificity of different astrocyte subpopulations with their specific genetic markers, thereby revealing their respective roles in injury, will provide a more precise indication to allow the targeting of specific astrocyte subpopulations for the treatment of CNS injuries. Such as the study of Hasel et al. (2021), in the mouse inflammation model, they divided astrocyte subgroups according to the difference between transcriptome and anatomical location and found that Cluster 8 is widely present in inflamed brains, but few in normal brains. In subsequent studies, treatment attempts can be made against Cluster 8 to inhibit the production of Cluster 8, or convert Cluster 8 into a neuroprotective or even neutral RAs subgroup to reduce inflammation. Although they have been proposed to express unique marker genes, little is known regarding the process involved in the transformation between RAs and SAs given that research attention has primarily focused on inflammation and glial scar formation after injury. In the absence of theoretical support, there is no way to talk about the treatment of targeted SA. As detailed in this review, clarifying how SAs are generated may provide ideal treatment and management options for CNS injuries. Based on the precise type of astrocytes, targeting harmful RA subgroups in the early stage of injury to reduce neuronal death and tissue destruction, and changing the extracellular matrix and reducing scar formation through the regulation of SA in the later stage to weaken the external inhibitory factors of nerve regeneration. This kind of treatment is worth looking forward to.

## Author Contributions

BN designed the research and revised the manuscript. YZ found some articles. GY wrote the article. All authors contributed to the article and approved the submitted version.

## Conflict of Interest

The authors declare that the research was conducted in the absence of any commercial or financial relationships that could be construed as a potential conflict of interest.

## Publisher’s Note

All claims expressed in this article are solely those of the authors and do not necessarily represent those of their affiliated organizations, or those of the publisher, the editors and the reviewers. Any product that may be evaluated in this article, or claim that may be made by its manufacturer, is not guaranteed or endorsed by the publisher.
